# Higher incidence of acute respiratory distress syndrome in cardiac surgical patients with elevated serum procalcitonin concentration: a prospective cohort study

**DOI:** 10.1186/s40001-020-00409-2

**Published:** 2020-03-30

**Authors:** Zhang-Bo Cheng, Han Chen

**Affiliations:** 1 Department of Cardiosurgery, Fujian Provincial Hospital, 134 Dongjie Street, Fuzhou, Fujian China; 2 Surgical Intensive Care Unit, Fujian Provincial Hospital, 134 Dongjie Street, Fuzhou, Fujian China; 3 Fujian Provincial Clinical College, Fujian Medical University, Fuzhou, Fujian China

**Keywords:** Procalcitonin, Acute respiratory distress syndrome, Cardiopulmonary bypass

## Abstract

**Background:**

Inflammatory response is activated during cardiopulmonary bypass (CPB), which may lead to acute respiratory distress syndrome (ARDS) and procalcitonin (PCT) increases during this inflammatory response. The objective of the study was to validate whether patients with higher serum PCT concentrations have a higher incidence of ARDS.

**Methods:**

The study was a prospective, single-center, observational cohort study. All patients who received cardiac surgery with CPB were screened for study eligibility. Patients were assigned to the PCT-elevated cohort or the control cohort according to serum PCT concentration on the first postoperative day with a cut-off value of 7.0 ng/mL. Patients were followed up until the 7th postoperative day. The primary endpoint was the incidence of ARDS, which was diagnosed according to the Berlin definition.

**Results:**

A total of 296 patients were enrolled, 64 patients were assigned to the PCT-elevated cohort and 232 patients were assigned to the control cohort. PCT concentration was 16.23 ± 5.9 ng/mL in the PCT-elevated cohort, and 2.70 ± 1.43 ng/mL in the control cohort (*p* < 0.001). The incidence of ARDS was significantly higher in the PCT-elevated cohort than in the control cohort (21.9% versus 5.6%, *p* < 0.001). The incidence of moderate-to-severe ARDS was also significantly higher in the PCT-elevated cohort than in the control cohort (10.9% versus 0.4%, *p* < 0.001). The hazard ratio of ARDS at 7 days in the PCT-elevated cohort, as compared with the control cohort, was 6.8 (95% confidence interval 2.7 to 17.4). The hazard ratio of moderate-to-severe ARDS in the PCT-elevated cohort was 57.3 (95% confidence interval 10.4 to 316.3). The positive predictive value of PCT for ARDS and moderate-to-severe ARDS were 0.242 and 0.121, respectively; the negative predictive value of PCT for ARDS and moderate-to-severe ARDS were 0.952 and 1.0, respectively.

**Conclusion:**

Cardiac surgical patients with elevated PCT concentration have a higher incidence of ARDS. Elevated PCT may serve as a warning signal of postoperative ARDS in patients undergoing cardiac surgery with CPB.

*Study registration* Chinese Clinical Trial Registry (ChiCTR-OCH-14005076)

## Background

Epidemiological studies have shown that cardiac surgery with cardiopulmonary bypass (CPB) is a known risk factor for acute respiratory distress syndrome (ARDS) [1–5]. Although being a highly sterile type of surgery, cardiac surgery with CPB can lead to a systemic inflammatory response with the activation of multiple inflammatory pathways involving both cellular elements and soluble proteins [6, 7]. The possible causes of the inflammatory response are the exposure of blood to non-physiological surfaces, ischemia–reperfusion injury due to aortic clamping and extracorporeal circulation [8], as well as translocation of endotoxins from gut to the bloodstream [9], which can activate inflammatory cascades similar to those observed in sepsis. Cytokines such as interleukin (IL)-6, IL-8, tumor necrosis factor, C-reactive protein, lipoprotein-binding protein and procalcitonin (PCT) may play an important role in the immune reaction.

PCT is initially described as an early, sensitive and specific marker for sepsis associated with bacterial infection [10]. However, it also increases in clinical situations without infections such as major surgery, burns, or trauma [11]. Previous studies suggested that the concentration of serum PCT increased at the end of CPB, reaching its peak on the first day and then declined rapidly [6, 12]. Previous studies also suggested that significant elevation of PCT level can be observed when complications (including pulmonary complications) presented [6, 13, 14]. However, ARDS was not differentiated from other kinds of pulmonary edema in these studies [6, 13, 14]. It is important to do so because patients undergoing cardiac surgery are at high risk of developing cardiogenic pulmonary edema, which is probably due to cardiac dysfunction or fluid overload, whereas the development of ARDS is predominantly attributed to the systemic inflammatory response. It is still unknown whether high PCT concentration indicates a high risk of developing ARDS. In the present study, our aim was to validate whether patients with higher PCT concentrations have a higher incidence of ARDS.

## Methods

### Ethical and informed consent

The present study was a prospective, single-center, observational cohort study in patients after elective cardiac surgery. The study protocol was approved by the Institutional Review Board of Fujian Provincial Hospital. The study was registered on August 8th, 2014 at the Chinese Clinical Trial Registry (ChiCTR-OCH-14005076). The study protocol was published in October, 2014 [15]. The major change of the protocol was that we used Kaplan–Meier methods in the statistical analysis to assess the difference in the incidence of ARDS. Written consent was obtained from each patient or an appropriate substitute decision-maker.

### Study setting and population

All patients who received cardiac surgery under CPB were screened for study eligibility.

Inclusion criteria were:

1. Age 18 years and above;
2. Programmed cardiac surgery under CPB;
3. Being free from active preoperative infection or inflammatory disease (meet all of the following criteria at study entry: leukocyte count < 12*10^9^/L, PCT < 0.5 ng/mL, body temperature < 37.5 °C);
4. Able to consent.

The time point of baseline PCT measurement was not strictly limited. Usually, PCT was measured together with the routine preoperative blood tests, which were taken on the next day after hospital administration.

Exclusion criteria were:

1. History of chronic obstructive pulmonary disease, asthma or interstitial lung disease;
2. History of lung surgery;
3. Pregnant or lactating women;
4. Unwillingness of the patient to provide consent;
5. Enrolled in another trial.

### Data collection and follow-up

At study entry, data on demographic, history of smoking, history of past illness characteristics, diagnosis were collected. The methods of anesthesia, CPB and perioperative management were detailed in the published protocol [15]. Type of surgery, duration of operation, CPB and aortic clamping and net fluid balance during the operation were recorded. Patients were transmitted to the ICU after the surgery. The serum concentration of PCT on the first postoperative day was recorded. Patients were assigned to the PCT-elevated cohort or control cohort according to serum PCT concentration on the first postoperative day with a cut-off value of 7.0 ng/mL.

Patients were followed up until the seventh postoperative day to determine whether the patient had developed ARDS. Blood gas analysis was tested at least once per day when the patients were in the ICU. After transferred to the routine room, blood gas analysis was tested when the physicians considered it was necessary, or when patients’ SpO_2_ cannot be maintained greater than 95% with a FiO_2_ of 0.5. ARDS was diagnosed according to the Berlin definition [16]. Two physicians made the diagnosis independently. Only the patients who were diagnosed ARDS by both physicians were considered of developing ARDS. Echocardiography or pulmonary artery catheter was used to exclude hydrostatic edema. Physicians who assess the development of ARDS were blind to patients’ PCT levels. N-terminal pro-B-type natriuretic peptide (BNP) was also tested on the first postoperative day. Daily vasoactive-inotropic score (VIS) [17] was calculated and the highest value was recorded. Patients were followed up until the 28th postoperative day to determine the occurrence of postoperative complications.

### PCT determination

Serum PCT levels were measured by a highly sensitive and specific commercially available immunoluminometric assay kit (Vidasbrahms PCT, mini VIDS, Italy) according to the manufacturer’s recommendation. Quantitative measurement allowed the determination of PCT concentrations ranging from 0.05 to 200 ng/mL.

### Study endpoints

The primary endpoint was the incidence of ARDS.

Secondary endpoints include:

1. The duration of mechanical ventilation;
2. The length of ICU stay;
3. Complications after surgery, including acute kidney injury, cardiac arrest, neurological dysfunction, myocardial infarction, reoperation and death from any cause.

### Statistical analysis

Categorical variables were presented as numbers and percentages and analyzed by χ^2^-test. Continuous variables were checked for normal distribution and presented as mean and standard deviation or median and inter-quartile range (IQR) as appropriate. Comparison of continuous variables was performed by using Student’s *t*-test for normally distributed variables and the Mann–Whitney *U* test for non-normally distributed variables. The incidence of ARDS was analyzed with the use of the Kaplan–Meier method and compared between cohorts with the use of the log-rank test. Multivariate logistic regression analysis was performed using forward procedures with factors showing a *p* value < 0.20 in univariate analysis. Receiver-operating characteristics (ROC) curve analysis was carried out to assess the predictive performance of PCT on the development of ARDS. All tests of significance were at the 5% significance level, and two-sided. Analyses were performed by using SPSS 23.0 (IBM Corporation, New York, USA) and GraphPad Prism 6 (GraphPad Software, California, USA).

## Results

**Fig. 1:**
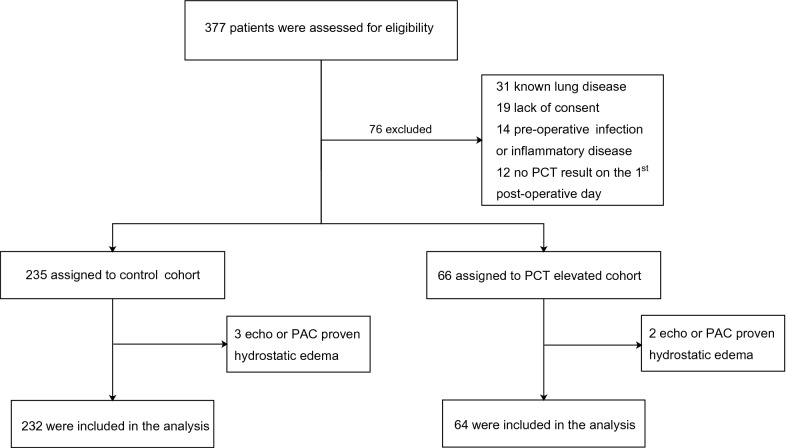
Outline of the screening and follow-up. *PCT* procalcitonin, *PAC* pulmonary artery catheter

We enrolled 296 patients between October 2014 and October 2017, 64 patients were assigned to the PCT-elevated cohort and 232 patients were assigned to the control cohort; 76 patients were excluded (Fig. 1). PCT concentration was 16.23 ± 5.9 ng/mL in the PCT-elevated cohort, and 2.70 ± 1.43 ng/mL in the control cohort (*p* < 0.001). There was no significant difference in demographic characteristics, disease history or type of surgery between the two cohorts. There was longer duration of operation (305.7 ± 63.5 min versus 264.4 ± 51.1 min, *p* < 0.001), CPB (147.2 ± 32 min versus 135.6 ± 22.3 min, *p* = 0.008) and aortic clamping (84.5 ± 17.6 min versus 78.4 ± 18.2 min, *p* = 0.017) in the PCT-elevated cohort than in the control cohort. There were significant correlations between PCT levels and both CBP and operation time (*r* = 0.430 and 0.528, respectively; all *p* < 0.001). There was no significant difference in intraoperative fluid balance or amount of transfusion between the two cohorts (Table 1).

**Table 1:** Demographic and surgical characteristics at baseline

	PCT elevated (*n* = 64)	Control (*n* = 232)	*p* value
Age (years, old)	61.0 ± 9.9	60.8 ± 10.3	0.904
Male, *n* (%)	41 (64.1)	119 (51.3)	0.070
Smoker, *n* (%)	14 (21.9)	52 (22.4)	0.927
Coexisting conditions			
Hypertension, *n* (%)	30 (46.9)	117 (50.4)	0.614
Diabetes mellitus, *n* (%)	18 (28.1)	65 (28)	0.986
Coronary artery disease, *n* (%)	52 (81.3)	176 (75.9)	0.364
NYHA grade III–IV, *n* (%)	34 (53.1)	132 (56.9)	0.590
Liver disease, *n* (%)	3 (4.7)	5 (2.2)	0.269
Preoperative drug therapy			
ACEI, *n* (%)	16 (25)	46 (19.8)	0.368
Beta-blocker, *n* (%)	21 (32.8)	71 (30.6)	0.735
Calcium channel blocker, *n* (%)	18 (28.1)	59 (25.4)	0.664
Aspirin, *n* (%)	32 (50)	99 (42.7)	0.296
Aspirin + clopidogrel, *n* (%)	20 (31.3)	56 (24.1)	0.249
Preoperative troponin I (ng/mL)	0.07 ± 0.04	0.08 ± 0.06	0.336
Preoperative Nt-pro-BNP (pg/mL)	826 ± 301	772 ± 294	0.201
Surgery type			0.080
CABG, *n* (%)	52 (81.3)	155 (66.8)	0.078
Valve replacement, *n* (%)	9 (14.1)	61 (26.3)	0.123
Other, *n* (%)^a^	3 (4.7)	16 (6.9)	> 0.999
Operation duration (min)	305.7 ± 63.5	264.4 ± 51.1	< 0.001*
CPB time (min)	147.2 ± 32	135.6 ± 22.3	0.008*
Aortic clamping time (min)	84.5 ± 17.6	78.4 ± 18.2	0.017*
Intraoperative fluid balance (mL)	124 ± 203	109 ± 229	0.626
RBC infusion (mL)	259 ± 316	183 ± 215	0.530
FFP infusion (mL)	294 ± 325	215 ± 228	0.306

*PCT* procalcitonin, *NYHA* New York Heart Association, *ACEI* angiotensin converting enzyme inhibitor, *Nt-pro-BNP* N-terminal pro-B-type natriuretic peptide, *CABG* coronary artery bypass grafting, *CPB* cardiopulmonary bypass, *RBC* red blood cell, *FFP* fresh frozen plasma

* *p* < 0.05

^a^Including adult congenital heart procedure, great artery surgery and combined surgery

**Fig. 2:**
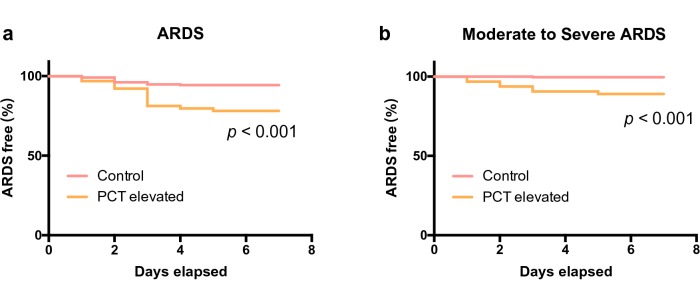
Kaplan–Meier survival estimates through postoperative day 7. **a** Probability of being ARDS free through postoperative day 7. **b** Probability of being free of moderate-to-severe ARDS through postoperative day 7

The PCT-elevated cohort had significantly lower P/F ratio than the control cohort (334 ± 88 versus 375 ± 56, *p* = 0.001). The incidence of ARDS was significantly higher in the PCT-elevated cohort than in the control cohort (21.9% versus 5.6%, *p* < 0.001; Fig. 2a). The incidence of moderate-to-severe ARDS (i.e., P/F ratio < 200) was also significantly higher in the PCT-elevated cohort than in the control cohort (10.9% versus 0.4%, *p* < 0.001; Fig. 2b). The hazard ratio of ARDS at 7 days in the PCT-elevated cohort, as compared with the control cohort, was 6.8 (95% confidence interval: 2.7 to 17.4). The hazard ratio of moderate-to-severe ARDS in the PCT-elevated cohort was 57.3 (95% confidence interval: 10.4 to 316.3). Among the ARDS patients, six patients were diagnosed with pneumonia (4 in the PCT-elevated cohort and 2 in the control cohort).

There were longer ICU stay and mechanical ventilation duration in the PCT-elevated cohort than in the control cohort (Table 2). There was no significant difference in mortality and renal failure rate between the two cohorts. There was also no significant difference in postoperative BNP, 1st-postoperative day fluid balance, white blood cell count and VIS between the two cohorts (Table 2).

**Table 2:** Outcomes data

	Overall population (*n* = 296)	PCT elevated (*n* = 64)	Control (*n* = 232)	*p* value
1st postoperative day fluid balance (mL)	672 ± 276	663 ± 248	727 ± 322	0.051
Nt-pro-BNP (pg/mL)	2699 ± 1373	2836 ± 1403	2662 ± 1365	0.372
PCT (ng/mL)	5.63 ± 6.34	16.23 ± 5.9	2.70 ± 1.43	< 0.001*
WBC (*10^9^/L)	13.6 ± 2.2	13.8 ± 2.8	13.5 ± 2.0	0.532
VIS	16.2 ± 9.6	16.0 ± 14.2	16.2 ± 8.0	0.898
P/F ratio	367 ± 66	334 ± 88	375 ± 56	0.001*
Severity of ARDS				< 0.001*
No ARDS, *n* (%)	269 (90.9)	50 (78.1)	219 (94.4)	< 0.001*
Mild ARDS, *n* (%)	19 (6.4)	7 (10.9)	12 (5.2)	0.576
Moderate ARDS, *n* (%)	7 (2.4)	6 (9.4)	1 (0.4)	< 0.001*
Severe ARDS, *n* (%)	1 (0.3)	1 (1.6)	0	0.864
MV duration (day)	1.7 ± 1.7	2.6 ± 3.0	1.4 ± 0.9	0.002*
ICU stay (day)	2.0 ± 2.4	1.7 ± 1.4	3.3 ± 4.3	< 0.001*
Mortality, *n* (%)	1 (0.3)	1 (1.6)	0 (0)	0.490
Renal failure, *n* (%)	4 (1.4)	2 (3.1)	2 (0.8)	0.165
Pneumonia, *n* (%)	6 (2.0)	4 (6.3)	2 (0.8)	0.027*

*PCT* procalcitonin, *Nt*-*pro*-*BNP* N-terminal pro-B-type natriuretic peptide, *WBC* white blood cell, *VIS* vasoactive-inotropic score, *ARDS* acute respiratory distress syndrome, *MV* mechanical ventilation

* *p* < 0.05

**Fig. 3:**
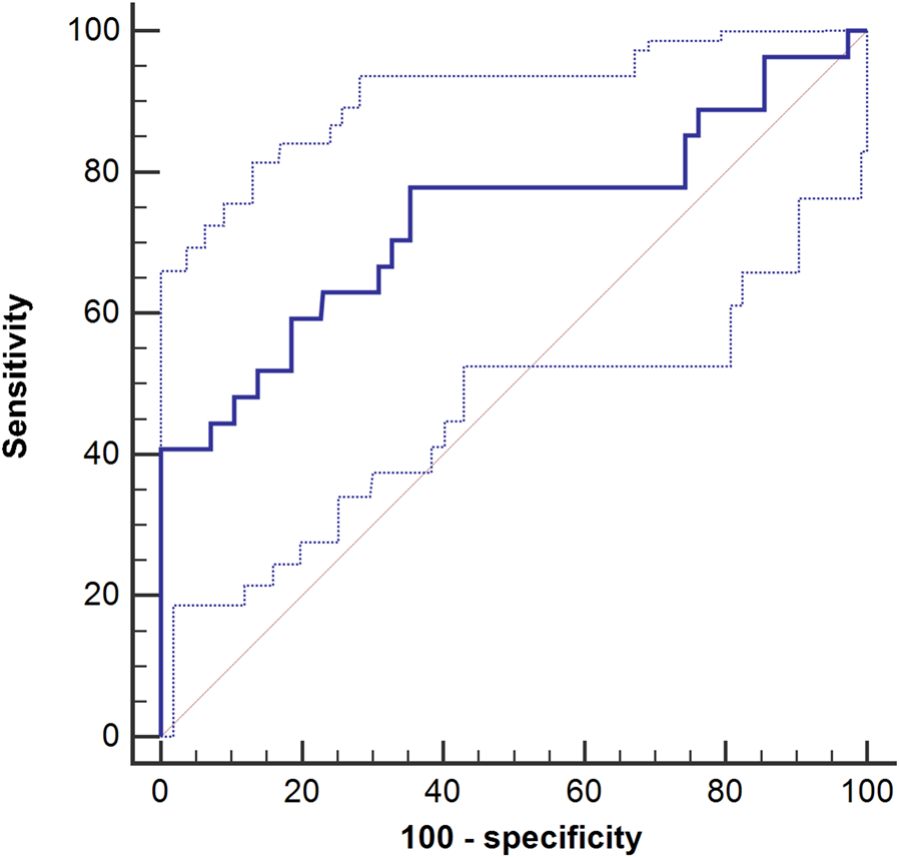
Receiver-operating characteristics curve assessing the predictive performance of PCT on the development of ARDS

After ROC analysis, PCT had an AUC of 0.734 (95% CI 0.680 to 0.783, *p* < 0.001) for ARDS prediction (Fig. 3). The optimal cut-off value of PCT was 6.5 ng/ml, yielding a sensitivity of 59.3% and a specificity of 81.4% for ARDS; whereas for moderate-to-severe ARDS, the sensitivity was 100% and specificity 79.9%. The performance of the ROC curve is summarized in Table 3.

**Table 3:** Value of PCT in predicting ARDS

	For ARDS	For moderate-to-severe ARDS
Sensitivity (95% CI)	59.3% (38.8%, 77.6%)	100% (63.1%, 100%)
Specificity (95% CI)	81.4% (76.2%, 85.9%)	79.9% (74.8%, 84.3%)
Positive predictive value (95% CI)	0.242 (0.145, 0.364)	0.121 (0.054, 0.225)
Negative predictive value (95% CI)	0.952 (0.916, 0.976)	1.0 (0.984, 1)
Positive likelihood ratio (95% CI)	3.19 (2.1, 4.8)	4.97 (3.9, 6.2)
Negative likelihood ratio (95% CI)	0.50 (0.3, 0.8)	< 0.01

Using a cut-off value of 6.5 ng/mL

*ARDS* acute respiratory distress syndrome

PCT level, age, gender, operation duration, CPB time, aortic clamping time and the fluid balance on the first postoperative day were included in multivariate logistic regression analysis. As a result of multivariate logistic regression analysis, only PCT concentration was extracted as an independent risk factor of ARDS (OR = 1.165, 95% confidence interval 1.106 to 1.226).

## Discussion

Although initially described as an early marker for sepsis associated with bacterial infection [10], PCT can also elevate after cardiac surgery that is considered highly sterile [6, 13, 14]. It has been shown that PCT liberation predominantly depended on the use of CPB in cardiac surgery [18]. It is also known that the development of ARDS is associated with the use of CPB [4]. However, it is still unknown whether high PCT indicates a higher incidence of ARDS. In this prospective cohort study, we provided data to support the hypothesis that patients with elevated PCT have a higher incidence of ARDS.

The pathophysiology of ARDS following CPB has not been completely defined yet. It is believed that the use of CPB is associated with the activation of multiple inflammatory pathways [7]. A few proinflammatory cytokines elevate in response to tissue injury, transfusion and endotoxic challenge, and lead to systemic proinflammatory status [19–22]. This systemic proinflammatory status results in neutrophil influx [22], pulmonary coagulopathy [22], capillary endothelial damage [23] and increased pulmonary vascular permeability [24], which are the characteristic findings in ARDS. In this case, an indicator of systemic inflammatory response might be helpful for alarming the development of ARDS. Although the mechanism has not yet been completely clarified, it is believed that bacterial translocation from the gastrointestinal tract and the associated release of endotoxin during CPB may play an important role in stimulating the liberation of PCT [18]. Therefore, PCT might serve as an indicator of the inflammatory response caused by CPB, and thereby warning the development of ARDS. Moreover, patients with higher PCT levels have significantly longer durations of surgery and CPB in our study, and (weak) positive correlations were also found. This is somehow explained by that a longer CPB time would lead to more proinflammatory cytokine release and a more profound inflammatory response. For instance, it has been reported that the magnitude of complement activation is proportional to the duration of CPB [25].

We used a cut-off value of 7.0 ng/mL for patient assignment in this study. This cut-off value was obtained from our previous retrospective case–control study in a similar study population, and more than 100 cases were enrolled in that study between 2010 and 2014 (unpublished data). Therefore, the present study was actually a prospective validation of elevated PCT as a predictor of ARDS. Using the data of this study we performed ROC curve analysis again and obtained an optimal cut-off value of 6.5 ng/mL. Two patients who were in the control cohort should be assigned to the PCT-elevated cohort if 6.5 ng/mL was chosen as the cut-off value, and the sensitivity would be slightly improved (51.9% to 59.3%) with no difference in specificity. Considering the tiny difference between the two cut-off values and the improved performance, we reported the performance of the ROC curve base on our new cut-off value (using the cut-off value of 6.5 ng/mL).

Our data suggest a very high negative predictive value of PCT. This means that we can use PCT as a tool to rule out patients who are unlikely to develop ARDS. However, our data suggest that the positive predictive value was not high for either ARDS or moderate-to-severe ARDS. This is probably because of the low morbidity of ARDS. In our study, the overall incidence of ARDS was 9.1%; this is comparable to the previous report (10.2%), which used the term “acute lung injury” [5]. The incidence of moderate-to-severe ARDS was 2.7%, which was also close to previous studies (that defined ARDS as a PaO_2/_FiO_2_ ratio < 200) [1, 2, 26]. Nevertheless, provided high mortality of ARDS, it is still good to have a tool to warn the possibility of developing ARDS, especially in those with risk factors such as massive transfusion, previous cardiac surgery, long-time CPB [2, 4], etc.

However, our patients had obviously lower mortality (1/27) compared with the overall ARDS population, who have a mortality of 20% to 50% [16]. The possible explanation is that ARDS is a heterogeneous syndrome that encompasses lung injury from both direct and indirect sources. A sepsis-like inflammatory response and endothelial damage caused by CPB leads to an extra-pulmonary ARDS, which is more homogeneous and recruitable. Therefore, in spite of severe reductions in oxygenation, these patients are less likely to develop ventilator-induced lung injury which worsens the lung injury and is associated with death. Once the inflammatory response ceases and the endothelial damage recovers, patients can rapidly recover. One more reason is that our patients were having elective surgery, which means that they had a better underlying health condition.

There were 6 patients who developed pneumonia, which happened after the sampling of PCT. This means that pneumonia was less likely be the reason for the elevation of PCT. However, the PCT-elevated cohort had a higher incidence of pneumonia as well. This is probably because of the longer duration of mechanical ventilation in the PCT elevation cohort, which is the risk factor of ventilator-associated pneumonia. Also, the pre-existing inflammatory lung edema may also attribute to the development of ventilator-associated pneumonia.

Although PCT has been used to predict postoperative pulmonary complications in patients undergoing cardiac surgery, ARDS was not differentiated from other kinds of pulmonary edema in previous studies [6, 13, 14]. It is important to distinguish ARDS patients because cardiac surgical patients are at high risk of developing cardiogenic pulmonary edema, whereas the development of ARDS is predominantly attributed to a systemic inflammatory response. In the present study, we used echocardiography or pulmonary artery catheter to rule out cardiogenic pulmonary edema (the numbers of patients diagnosed by echocardiography were 43 [67.2%] in the PCT-elevated cohort and 165 [71.1%] in the control cohort). This is an important strength of this study. We also measured Nt-pro-BNP as an indicator of cardiac function. The possible mechanism of production of BNP is the increase in regional ventricular wall stretch due to local depression of myocardial contraction [27]. There was no difference in BNP between the two cohorts in this study, suggesting a comparable cardiac function between cohorts. To the best of our knowledge, this is also a study with the largest sample size to investigate the value of PCT in predicting post-cardiosurgical ARDS.

### Limitations of the study

First, other proinflammatory factors such as IL-6, IL-8 or tumor necrosis factor were not measured in this study. Therefore, we were not able to confirm the relationship among CPB, systemic inflammatory response and elevated PCT. Second, we assessed cardiac function by using either echocardiography or pulmonary artery catheter which basically depended on the availability of pulmonary artery catheter—if there was a pulmonary artery catheter in place, we used it; if not, echocardiography was used. In this way we were not able to directly compare cardiac function between cohorts because we had complete data of neither echo nor pulmonary arterial wedge pressure. Instead, we used BNP as an indicator of cardiac function. Third, this study excluded patients with underlying pulmonary diseases who are at risk for ARDS and included only those who underwent elective CBP surgery. Therefore, the conclusion should be extrapolated with caution. Fourth, there was an imbalance in the number of patients between groups: the number of patients in the PCT-elevated cohort was about one-quarter to the number of patients in the control cohort. Finally, the specificity and sensitivity values of PCT predicting ARDS were of low statistical power. Predictions should not be drawn solely from PCT results.

## Conclusions

Cardiac surgical patients with elevated PCT concentration have a higher incidence of ARDS. Elevated PCT may serve as a warning signal of postoperative ARDS in patients undergoing cardiac surgery with CPB.

## Data Availability

The datasets used and/or analyzed during the present study are available from the corresponding author on reasonable request.

## Abbreviations

CPB: Cardiopulmonary bypass
ARDS: Acute respiratory distress syndrome
PCT: Procalcitonin
IL: Interleukin
Nt-pro-BNP: N-terminal pro-B-type natriuretic peptide
VIS: Vasoactive-inotropic score
IQR: Interquartile range
